# Supplementary material for: Chromothripsis during telomere crisis is independent of NHEJ, and consistent with a replicative origin
Source: Genome Res. 2019 May;29(5):737–49. doi: 10.1101/gr.240705.118 (PMC6499312; doi:10.1101/gr.240705.118)
Supplement: Supplemental Material [file supp_gr.240705.118_Supplemental_file_1.zip › contigs/annotated_contigs/DB46/contig.2.DB46_length_563_mean_cov_6.8809946714.docx]

**DB46_length_563_mean_cov_6.8809946714**

```
CTAAAGTCTAGAGGTAGGCAGGCCCAGTTGGTTCACAGATTGAACAATGACCTCAGAGACACTGGATTTTTACCCTCTCCCATTCTGCC
 >chr5:111318252-111318488 + E=2e-127
ATCTTTATCAGATTGATTTACCACTTCAGAGTTTCCAGATAGCTACAATAGCCACAAGCATCCTCTTAGGACAGCAACCAAGACAGAAG

AAATGGAAAGGGCAAAACAGATATTTTCTTATGAAATTCTTTATCATGAAAAAAAA|GT|GTTGAGGGAGGGTAGGAATGTAGGAAAGA
                                                         >chr5:111319108-111319437 + E=
AAGGAGTGCTAAGGATACAATTAGTTCAATATTGGGCATATTGAAAGTAAAGTGTCTGTTATTTCAAGGTTGAGGTATCTAAAAGACTT
2e-186
ACCAGTCTAGAGTTCAGAAAAGAAGTAAACTGCATTCTTTCTAGGACATTTTTTAGAACAGGTGGCTACTAAGAATTTCCCCAGGAAAT

TGGCTTAAACTGGAAATGTCCCAAGAAACTACTATCTATGGGGATGATGAAATCATGAATCGGAGGTTGTTCTGTGAGATGCTTAAAAT

ACAAAGAAAAGTAAATATCTGCACCTTGAGA
```
